# What is an appropriate material to use with a covering technique to prevent the recurrence of spontaneous pneumothorax?

**DOI:** 10.1186/1749-8090-9-74

**Published:** 2014-04-29

**Authors:** Hidetaka Uramoto, Fumihiro Tanaka

**Affiliations:** 1 Second Department of Surgery, School of Medicine, University of Occupational and Environmental Health, Kitakyushu 807, Japan; 2 Second Department of Surgery, School of Medicine, University of Occupational and Environmental Health, 1-1 Iseigaoka, Yahatanishi-ku, Kitakyushu 807-8555, Japan

**Keywords:** Spontaneous pneumothorax, Covering technique, Postoperative recurrence, Risk factor

## Abstract

**Background:**

The purpose of this retrospective study was to identify an appropriate material that can be used as a covering for patients with a spontaneous pneumothorax (SP). A total of 279 patients were studied over a period of eight years.

**Methods:**

The patient characteristics, surgical details and perioperative outcomes were analyzed. We compared the clinicopathological characteristics between recurrent and non-recurrent cases, and examined the associations with the material used for covering the SP, such as polyglycolic acid (PGA) sheets, a fibrinogen-based collagen fleece (TachoComb; TC) or regenerated oxidized cellulose mesh (ROCM).

**Results:**

The differences in the gender, smoking habits, lesion site, location, comorbidities, ipsilateral spontaneous pneumothorax (ISP), contralateral spontaneous pneumothorax (CSP) and surgery for ISP did not reach statistical significance between the patients treated with a covering of ROCM and those treated with PGA/TC, although the age of the patients was significantly different in these groups, with the ROCM group having younger patients (p = 0.024). The length of the operation was significantly shorter in the ROCM group (mean: 76.7 minutes) than in the PGA/TC cases (130.4 minutes, p = 0.015). Concerning the intraoperative factors, there were no significant differences with regard to the approach, buttress stapling, covering or surgeon. No postoperative recurrence was observed in this series. There were no significant differences in the perioperative outcomes. However, the drainage period was shorter in subjects who underwent covering with the ROCM (mean: 1.125 days) than with the PGA/TC (2.412 days, p = 0.030). Further, the hospital stay had a tendency to be shorter in subjects who underwent covering with ROCM than with PGA/TC.

**Conclusions:**

ROCM might be superior to PGA/TC as a material for covering SP in terms of the length of the operation and the drainage period. ROCM might decrease the hospital stay and the postoperative recurrence. Prospective studies in a larger cohort of patients will be necessary to define the optimal surgical technique to suppress the recurrence of SP.

## Background

Spontaneous pneumothorax (SP) remains a significant health problem, even when surgical management is performed. The recurrence rate has been estimated to range from approximately 10 %–20 % [1-3]. Bullae neogenesis often occurs on one side and at the edge of the suture line, creating a thin membrane that rises at an acute angle. Regenerated bullae are suspected to be created soon after thoracoscopic surgery, growing within a short time period, and finally rupturing within several months. This may be one of the major factors leading to postoperative recurrence [1,3]. Therefore, reinforcement of the visceral pleura around the staple line has been used in clinical practice [1]. Nevertheless, the best material to cover SP has not yet been established, and the results are not satisfactory at present.

Regenerated oxidized cellulose mesh (ROCM) was originally developed as an absorbable hemostatic material. Recently, there has been growing acceptance of the use of ROCM as a covering material for SP, with the primary objective of relapse prevention, because ROCM coverage could significantly decrease air leakage from pleural tears in pigs [4]. However, no human clinical experiences have been published so far. The purpose of this study was to compare the postoperative recurrence rates among patients treated with various materials for covering a spontaneous pneumothorax.

## Materials and methods

### Patients and clinicopathological features

The institutional review board of University of Occupational and Environmental Health approved this study. From 2005 to 2012, 279 patients requiring surgery due to SP were included in this retrospective study conducted at the University of Occupational and Environmental Health. Preoperative investigations included chest radiography and a high-resolution computed tomographic (CT) scans of the thorax. Six patients with ipsilateral lung cancer and one with lymphangioleiomyomatosis (LAM) were excluded from the analyses because their disease state was very complicated compared with that of the patients with SP. Finally, a total of 272 patients were included in the present series. The indications for surgical treatment of patients with SP included a persistent air leak, tension pneumothorax, obvious existence of bulla lesions at first presentation, hemopneumothorax, the occupation of the patient and cases of ipsilateral or contralateral recurrences. The data were collected retrospectively for all patients, and included a detailed history, age, sex, smoking habits, past history of episodes of pneumothorax, treatment modalities and surgical details. The median age of the patients was 28 years old. The postoperative variables assessed included the use of pleurodesis, postoperative complications, duration of chest drainage and the length of the hospital stay. The patients were discharged from the hospital after surgery, and the follow-up data were collected via the outpatient department.

#### Surgical procedures and management

All patients underwent general anesthesia and single lung ventilation with a double-lumen endotracheal tube and were placed in the lateral decubitus position. The 5- or 10-mm 0° video thoracoscope was inserted in the 7th intercostal space in the midaxillary line, making use of the hole made for the preoperative drainage tube. After inspection of the thoracic cavity, one or two additional ports were placed. The 5- or 10-mm working ports were placed in the 5th intercostal space between the scapula tip and the anterior axillary line. In principle, the blebs were grasped with an endograsper and were excised with the endo-GIA stapling device (Auto Suture Company Division, United States Surgical Corporation, Norwalk, USA) or ECHELON device (Ethicon Endo-Surgery, Inc; Cincinnati, Ohio) or were hand stitched. Buttress stapling was performed using polyglycolic acid (PGA) felt (Neoveil, Gunze Ltd, Kyoto, Japan), DUET TRS (Covidien Autosuture, Mansfield, MA, 131 USA) or absorbable polyglycolic acid (PGA): trimethylene carbonate (SEAMGUARD: WL. Gore & Associates, Inc., Flagstaff, Arizona, USA). z (Beriplast P; Marburg, Germany or Bolheal: The Chemo-Sero-Therapeutic Research Institute, Kumamoto, Japan) or ROCM (Surgicel; 2 x 3 inch: Johnson & Johnson, New Brunswick, NJ, USA) with only 5 ml of autologous blood. An air leak test was performed under a pressure loading of 20 cm H_2_O. A chest tube was placed through one of the port sites. The surgery was performed by a surgical team including one surgical consultant, and procedures were selected based on the intraoperative findings.

#### Postoperative management and follow-up

All patients were extubated in the operating room. The chest tube was connected to an aspiration system, and negative suction of 5 or 10 cmH_2_O was applied. Intercostal drains were removed when the underlying lung was expanded with no residual air leaks. Postoperative pain was primarily controlled by means of a thoracic epidural block, and oral analgesic medication was administered when necessary. The patients were discharged from the hospital when they were fully mobile. A follow-up was conducted for all patients. The mean follow-up and relapse-free periods were 287 days (ROCM: 52, PGA/TC: 297) and 366 days, respectively.

#### Statistical analyses

Categorical variables were evaluated using the chi-square test and the t-test was utilized to analyze continuous variables between the two groups. Differences were considered to be statistically significant for p-values < 0.05. The data were analyzed using the StatView software package (Abacus Concepts, Inc., Berkeley, CA).

## Results

### The relationships between recurrence and the clinicopathological characteristics

In all, 272 patients were studied in this retrospective review. No covering technique was used in 63 cases. Twelve and 8 patients, respectively, underwent hand stitching for blebs, and device- plus hand stitching. Three cases underwent only searching and abrasion. A covering technique without stapling was used in one case. Therefore, 185 cases were evaluated in a definitive fashion.

The clinicopathological characteristics of the patients are shown in Table 1. All of the patients were Japanese, and they consisted of 159 males and 26 females in this series, with a mean age of 38.2 years (range 14–94 years). The number of patients with and without a smoking habit was 70 and 115, respectively. Ninety-four and 91 cases were right- and left-sided SP, respectively. Of these patients, one hundred and fifty-four (83.2 %) had SP located on the top of the lung. Twenty-nine (15.7 %) patients had a comorbid condition. One hundred and thirty-four were first-episode patients. We compared the clinicopathological characteristics between the cases covered with the ROCM and those covered with PGA/TC. The differences between the groups in the gender, smoking habit, lesion site, location, comorbidities, ISP, CSP and surgery for ISP did not reach statistical significance. However, the ROCM was used more often in the younger patients. This might have been due to (1) the careful administration of fibrin glue in combination with ROCM in a patient and (2) the uncertainties for long-term safety of developing an infection by fibrin glue itself. The length of the operation was also shorter in the ROCM group than in the PGA/TC group.

**Table 1:** The relationship between the material used for covering SP and the clinicopathological characteristics

**Characteristic**	**Total**	**ROCM**^**a**^	**%**	**PGA/ TC**^**b**^	**%**	***p*-value**
All cases	185	8	4.3	177	95.7	
Age (years)						
<28	90	7	7.8	83		
≥28	95	1	1.1	94		0.024
Gender						
Male	159	8	5.0	151		
Female	26	0	0.0	26		0.242
Smoker						
Yes	70	2	2.9	68		
No	115	6	5.2	109		0.444
Lesion site						
Right	94	4	4.3	90		
Left	91	4	4.4	87		0.962
Lesion location						
Top	154	6	3.9	148		
Other than top	31	2	6.5	29		0.523
Comorbidities						
Yes	29	0	0.0	29		
No	156	8	5.1	148		0.213
ISP^c^						
Yes	51	1	2.0	50		
No	134	7	5.2	127		0.330
CSP^d^						
Yes	42	3	7.1	39		
No	143	5	3.5	138		0.307
Surgery for ISP						
Yes	17	0	0.0	17		
No	168	8	4.8	160		0.358
Length of operation (min)		76.7 (40–160)		130.4 (35–315)		0.015

^a^ROCM: regenerated oxidized cellulose mesh, ^b^PGA: polyglycolic acid/ TC: TachoComb, ^c^ISP: ipsilateral spontaneous pneumothorax, ^d^CSP: contralateral spontaneous pneumothorax.

### Surgical data and perioperative results

We also compared the clinicopathological characteristics between recurrent and non- recurrent cases. There were 23 (12.4 %) postoperative recurrences. One hundred and seventy (91.9 %) subjects underwent video-assisted thoracoscopic surgery (VATS), while an open thoracotomy was carried out in 15 patients. Concerning the intraoperative factors, there were no significant differences in the approach, buttress stapling, covering, surgeon or length of the operation. The differences in intraoperative factors did not reach statistical significance between the two groups. The indications for fibrin glue were comprehensively decided based on the age of the patient, parenchyma of the lung and the degree of the air leak. Only one case in the ROCM group was treated using fibrin glue. On the other hand, 12 among the 177 cases in the PGA/TC group were treated using fibrin glue (p < 0.001). There were no significant differences in the perioperative recurrence based on the use of fibrin glue. The incidence of postoperative recurrence in the cases covered with PGA and TC was 14.2 and 10.0 %, respectively. However, no postoperative recurrence was observed in the ROCM series (Table 2). There were no significant differences in the perioperative outcomes except for the drainage period (Table 3). The drainage period was significantly shorter in subjects who underwent covering with ROCM than with PGA/TC. Furthermore, the hospital stay tended to be shorter in subjects who underwent covering with ROCM than with PGA/TC. A univariate or multivariate analysis of the factors contributing to the recurrence could not be performed, because the incidence of recurrence in the ROCM group was zero.

**Table 2:** The relationship between recurence and the intraoperative factors

**Characteristic**	**Total**	**Rec**^**a**^	**%**	**Non-rec**	**%**	***p*-value**
	185	23	12.4	162	87.6	
Approach						
VATS	170	21	12.4	149		
Thoracotomy	15	2	13.3	13		0.912
Buttress stapling						
Yes	24	4	16.7	20		
No	161	19	11.8	142		0.500
Covering						
ROCM	8	0	0.0	8		
PGA/ TC	177	23	13.0	154		0.276
Surgeon						
Consultant	88	13	14.7	75		
Trainee	97	10	10.3	87		0.358
Length of operation (min)		133.4 (40–280)		127.3 (35–315)		0.661

^a^postoperative recurrence.

**Table 3:** The periopetative outcomes

**Characteristic**	**Total**	**ROCM**	**%**	**PGA/ TC**	***p*-value**
Pleurodesis					
Yes	14	0	0.0	14	
No	171	8	4.7	163	0.408
Postoperative complications					
Yes	6	0	0.0	0	
No	179	8	4.5	171	0.597
Drainage period (days)		1.125 (1–2)		2.412 (1–15)	0.030
Hospital stay (days)		3.8 (3–6)		6.2 (2–31)	0.116

## Discussion

The present study clearly demonstrated three major findings. First, the length of the operation was shorter in the ROCM group than in the PGA/TC group. This finding is reasonable, because PGA/TC generally requires the use of fibrin-glue, and it takes several minutes to dissolve the powder. On the other hand, covering by the ROCM was performed more rapidly using autologous blood from the artery line as continuance monitor.

Second, concerning the intraoperative factors, there were no significant differences in the approach, buttress stapling or surgeon between the ROCM and PGA/TC cases. Nevertheless, while recurrence rates of around 10 % were seen in the PGA/TC cases, no postoperative recurrence was observed in the ROCM series. A recent animal study revealed that covering tissue with ROCM prevents a long-term foreign body reaction and the development of adhesions at the surgical site, and also increases mesothelium-like cell proliferation [5]. These results suggest that ROCM may prevent pleural adhesions and strengthen the visceral pleura by increasing its thickness [6]. Thus, ROCM might be the most effective covering material, at least partly due to its prevention of adhesion to the chest wall and its providing thickness to the visceral pleura [3]. Therefore, the use of ROCM is a rational approach, because PGA sheets can allow serious adhesion to the parietal pleura to occur within a few weeks after surgery [7]. Furthermore, the postoperative recurrence mainly attributed to bullae neogenesis near the suture line is thought to result from the lung being roughly picked up with a stapler during thoracoscopic surgery [7]. Therefore, it is considered to be more important to solve the problems associated with the residual lung surface than those associated with the parietal pleura. Interestingly, successful experiences with surgical treatment by using ROCM for patients with alpha-1 antitrypsin deficiency, LAM, Birt–Hogg–Dubé syndrome (BHD) and chronic obstructive pulmonary disease (COPD), which is associated severe emphysema and pneumothorax, have been reported [6-9].

Third, the drainage period was significantly shorter in subjects who underwent covering with the ROCM than with PGA/TC. The hospital stay also had a tendency to be shorter in subjects who underwent covering with ROCM than with PGA/TC. The reason why there was no significant difference in the hospital stay, which generally correlates with the drainage period, might be due to the existence of comorbidities other than SP and the small number of patients treated with the ROCM.

We believe that there are several potential advantages associated with the ROCM, without any apparent disadvantages. (1) The length of the operation and drainage periods were significantly shorter in patients evaluated using the conventional methods. This result can provide major benefits for the patients, because it allows for minimally-invasive surgery and decreases the cost of medical care. (2) It is easy to perform, even when a reoperation is being performed. (3) It takes just a few minutes to perform. (4) The new method does not require fibrin-glue, which might increase the risk of infection, and increases the costs of the operation, because the fibrin-glue is expensive. (5) This technique might be useful not only for SP, but also for cases of limited resections, such as wedge resection and segmentectomy, for lung tumors. (6) This technique is inexpensive, and the ROCM costs just $11.30 US. On the other hand, the PGA sheet and TC cost $92.60 US and $303.90 US, respectively.

However, the present study is associated with some limitations that should be kept in mind when interpreting the results: (i) its retrospective nature, (ii) the fact that it was carried out at a single institution, (iii) there were imbalances in the patients’ characteristics that cannot be excluded given the short periods and small number of patients treated with the ROCM, (iv) university hospitals have a tendency to collect cases with high comorbidities. Nevertheless, the results address an important issue, since proper covering can improve the surgical outcome. To overcome these limitations, prospective studies in a larger cohort of patients will be necessary to clarify the risk factors for recurrence and to define the optimal surgical technique to suppress the recurrence of SP [10].

## Conclusions

The current results revealed that the ROCM is an appropriate material for covering SP, and might be superior to PGA/TC with regard to shorting the length of the operation and the drainage period.

## Abbreviations

SP: Spontaneous pneumothorax; ROCM: Regenerated oxidized cellulose mesh; ISP: Ipsilateral spontaneous pneumothorax; CSP: Contralateral spontaneous pneumothorax; CT: Computed tomography; LAM: Lymphangioleiomyomatosis; PGA: Polyglycolic acid; TC: TachoComb; VATS: Video-assisted thoracoscopic surgery; BHD: Birt–Hogg–Dubé syndrome; COPD: Chronic Obstructive Pulmonary disease.

## Competing interests

Dr. Uramoto and Tanaka have no competing interests and conflict of interest or financial ties to disclose.

## Authors’ contributions

This report reflects the opinion of the authors and does not represent the official position of any institution or sponsor. The contributions of each of the authors were as follows. HU were responsible for reviewing previous research, journal hand searching, and drafting report. FT was responsible for project coordination. All authors have read and approved the final manuscript.

## References

[B1] Muramatsu T Nishii T Takeshita S Ishimoto S Morooka H Shiono M Preventing recurrence of spontaneous pneumothorax after thoracoscopic surgery: a review of recent results Surg Today 2010 9 696 699 10.1007/s00595-009-4208-1 20676850

[B2] Uramoto H Tanaka F Natural air leak test without submergence for spontaneous pneumothorax J Cardiothorac Surg 2011 9 165 10.1186/1749-8090-6-165 22196849 PMC3259056

[B3] Kurihara M Kataoka H Ishikawa A Endo R Latest treatments for spontaneous pneumothorax Gen Thorac Cardiovasc Surg 2010 9 113 119 20349299 10.1007/s11748-009-0539-5

[B4] Luh SP Chou HH Tsai TP Chen JY Chou MC Wang YH Lee CJ Effect of Surgicel coverage with topical electrocauterization for preventing and sealing pulmonary air leakage Int Surg 2004 9 190 194 15730097

[B5] Biçer M Bayram AS Gürbüz O Senkaya I Yerci O Tok M Anğ E Moğol EB Saba D Assessment of the efficacy of bio-absorbable oxidized regenerated cellulose for prevention of post-operative pericardial adhesion in the rabbit model J Int Med Res 2008 9 1311 1318 10.1177/147323000803600619 19094441

[B6] Kusu T Nakagiri T Minami M Shintani Y Kadota Y Inoue M Sawabata N Okumura M Null allele alpha-1 antitrypsin deficiency: case report of the total pleural covering technique for disease-associated pneumothorax Gen Thorac Cardiovasc Surg 2012 9 452 455 22544422 10.1007/s11748-012-0015-5

[B7] Kurihara M Kataoka H Ebana H Endo R Chemical pleurodesis should absolutely be avoided to prevent LAM patients from recurrent pneumothorax considering lung transplantation (clinical and experimental study of total pleural covering technique) 2008 Cincinnati, OH: Presented at the LAM Symposium

[B8] Noda M Okada Y Maeda S Sado T Sakurada A Hoshikawa Y Endo C Kondo T A total pleural covering technique in patients with intractable bilateral secondary spontaneous pneumothorax: Report of five cases Surg Today 2011 9 1414 1417 10.1007/s00595-010-4427-5 21922367

[B9] Lee JM Pan JD Lin WC Lee YC Using Surgicel to buttress the staple line in lung volume reduction surgery for chronic obstructive pulmonary disease J Thorac Cardiovasc Surg 2006 9 495 496 10.1016/j.jtcvs.2005.09.039 16434295

[B10] Uramoto H Shimokawa H Tanaka F What factors predict recurrence of a spontaneous pneumothorax? J Cardiothorac Surg 2012 9 112 10.1186/1749-8090-7-112 23075329 PMC3488480

